# Open-target sparse sensing of biological agents using DNA microarray

**DOI:** 10.1186/1471-2105-12-314

**Published:** 2011-07-29

**Authors:** Mojdeh Mohtashemi, David K Walburger, Matthew W Peterson, Felicia N Sutton, Haley B Skaer, James C Diggans

**Affiliations:** 1 Emerging & Disruptive Technologies, The MITRE Corporation, McLean, Virginia, USA; 2 MIT CSAIL, Cambridge, MA, USA

## Abstract

**Background:**

Current biosensors are designed to target and react to specific nucleic acid sequences or structural epitopes. These 'target-specific' platforms require creation of new physical capture reagents when new organisms are targeted. An 'open-target' approach to DNA microarray biosensing is proposed and substantiated using laboratory generated data. The microarray consisted of 12,900 25 bp oligonucleotide capture probes derived from a statistical model trained on randomly selected genomic segments of pathogenic prokaryotic organisms. Open-target detection of organisms was accomplished using a reference library of hybridization patterns for three test organisms whose DNA sequences were not included in the design of the microarray probes.

**Results:**

A multivariate mathematical model based on the partial least squares regression (PLSR) was developed to detect the presence of three test organisms in mixed samples. When all 12,900 probes were used, the model correctly detected the signature of three test organisms in all mixed samples (mean(*R^2^*)) = 0.76, CI = 0.95), with a 6% false positive rate. A sampling algorithm was then developed to sparsely sample the probe space for a minimal number of probes required to capture the hybridization imprints of the test organisms. The PLSR detection model was capable of correctly identifying the presence of the three test organisms in all mixed samples using only 47 probes (mean(*R^2^*)) = 0.77, CI = 0.95) with nearly 100% specificity.

**Conclusions:**

We conceived an 'open-target' approach to biosensing, and hypothesized that a relatively small, non-specifically designed, DNA microarray is capable of identifying the presence of multiple organisms in mixed samples. Coupled with a mathematical model applied to laboratory generated data, and sparse sampling of capture probes, the prototype microarray platform was able to capture the signature of each organism in all mixed samples with high sensitivity and specificity. It was demonstrated that this new approach to biosensing closely follows the principles of sparse sensing.

## Background

To date, most biosensors can be considered to be 'target-specific' systems in that their detection elements are built to respond to a fixed number of organisms, and are designed to be non-responsive in the absence of those organisms. In fielded sensors, PCR-based technologies are often selected for their specificity and low per-assay cost. While this targeted approach is very effective in an environment where specific biological events are expected, a biosensing infrastructure capable of rapidly responding to new or engineered biological threats while maintaining a low cost of operation requires increased flexibility. Targeted platforms, like those using specific PCR primers for qualitative or quantitative amplification for detection, require creation of new physical capture reagents when new organisms are targeted [1]. These platforms are also often limited in the total possible number of parallel assays run at any one time as multiplexing tens or hundreds of PCR reactions greatly increases assay complexity. To mitigate the limitations of such approaches, there have been previous efforts to design high-density microarrays that are representative of groups or families of organisms and while these sensors would likely still offer information for novel threats, assured classification at the species or strain level would be impossible without re-engineering and re-deployment of sensing devices [2-4].

Microarray-based detection and identification approaches often consist of a series of probes designed with particular target genomes in mind; if a probe hybridizes, the analyst can be reasonably sure the organism or target represented by that probe is present in the original sample. In some cases, multiple probes can be used to create 'fingerprints' representative of particular organisms, but this requires a great deal of up-front probe design effort [5] such as assuring specificity of probe sequence and lack of cross-hybridization. This approach has been used previously to detect viruses [2,3,6,7]; in one example by designing 70-mer probes unique to each of more than 100 viral species [2]. Microarrays with species- or strain-specific probes have also been designed to differentiate between strains of *Staphylococcus aureus *by generating lists of thermodynamically-favorable probes from regions of sequence unique to particular strains [8-10]. Additional efforts have also constructed systems for the design of probes specific at the level of individual gene families [10], recognizing that some of these families will be specific for related pathogens.

While these approaches achieve an increase in robustness by using multiple, parallel measurements for each target organism, they still rely upon *a priori *knowledge of agent sequence. They are also limited in the scope of intended detection capability to only those organisms for which the individual arrays have been explicitly designed. However, the constraints placed on probes generated to match unique sequence regions in a family of organisms, by definition limit the capacity for these probes to hybridize to distinct novel or engineered organisms. An open-target design would provide data regardless of whether a particular biological event was expected, thus allowing new microorganisms to be recognized, characterized and managed in short order.

One presumed drawback in the design of an open-target biosensor, however, is that the greater the number of biological species to be detected, the larger the array size required. Thus, to detect the presence of even a few microorganisms, conventional wisdom dictates that the microarray would have to be very large to capture distinct genomic patterns with high degree of specificity, an endeavour that is not cost effective in environmental monitoring.

It has recently been suggested that many natural phenomena are sparse in that they can be represented in a compressed format using the proper basis [11-16]. Sparsity denotes that, to recover a signal of interest, the number of degrees of freedom needed to approximate the signal may, in principle, be much smaller than the length of the signal. This is the foundation for the new theory of sparse, or compressive sensing (CS) [13-15]. The main principle of CS is that for a signal x of length *N*, if x is *K*-sparse in some basis (*K *<<*N*), which implies that it has *K *non-zero entries and *N-K *zero elements, then M linear measurements of x suffice to recover the signal, *M  *<*N *and *M *=*O*(*K*log(*N/K*)). Let y be the vector of *M *measurements of x. Then in matrix notation we have y = Φx. The key challenge in this framework lies in the design of a *M *× *N *sensing matrix Φ, which together with y and the sparsity condition imposed on x, would be capable of accurate recovery or detection of x. For CS to apply, in addition to the constraint that x must be sparse, the sensing matrix must satisfy the restricted isometry property (RIP) [15] which implies that the rows of Φ should be incoherent with respect to the signal sparsity basis.

Recently, Dai et al. have proposed that DNA microarrays can be designed using the notion of CS [17]. They used the NCBI Clusters of Orthologous Groups (COG) database, which contains orthologous sets of proteins from 66 organisms corresponding to conserved protein domains. Challenges of this approach include how to translate protein back to less conserved DNA sequences and species which lack certain clustered proteins. Species which DNA encode these proteins differently than the array probe sequences would also not be detected.

In this paper, we put forward the notion that an open-target design is a viable approach to biosensing based on the principle of sparsity. Using laboratory-generated data, we provide strong evidence that: First, the underlying genomic imprints of multiple biological organisms can be captured succinctly using a small codebook, or collection of microarray probes, not specifically designed to respond to the target organisms. And second, our design approach follows closely the principles of sparse sensing, and thus CS is an applicable and sensible notion for biological sensing.

## Methods

### Microarray Probe Design

Potential probe sets were generated using Variable-length Markov Chains (VLMCs) [18], implemented using the *vlmc *package in the R [19] software environment. VLMCs were trained on genomic sequences from seven prokaryotic pathogens, listed in Table 1, and then used to emit 25-mer sequences for use as microarray probes. A sequence length of 25 had been previously identified as a good trade-off between hybridization specificity and diversity [20]. Genomic sequences were downloaded from the NCBI Genomes database in GenBank [21], and are described in Table 1.

**Table 1:** Pathogenic Sequences

Species	Pathogenicity	GenBank ID
Bacillus anthracis (Ames strain)	Anthrax	NC_003997

Yersinia pestis (CO92)	Bubonic plague	NC_003143

Francisella tularensis (Schu 4)	Tularemia	NC_006570

Brucella suis	Brucellosis	NC_004310

Burkholderia mallei	Glanders	NC_006348

Burkholderia pseudomallei	Melioidosis	NC_006350

Escherichia coli O157 H7 str. Sakai	Hemolytic uremic syndrome	NC_002695

Genomes retrieved from Genbank were used in the VLMC model to generate probes.

To investigate the impact of sequence sampling lengths and strategies on the final probe design, VLMCs for three different training sets were used to generate probes by sampling:

• 500 bp from each of 7 genomic sequences, resulting in a total of 3,500 long input sequence

• 5000 bp from each of 7 genome sequences, resulting in a total of 35,000-long input sequence

• 12,000 bp from each of 3 of the 7 sequences (identified in bold in Table 1), resulting in 36,000-long input sequence

Samples were taken randomly from each genome without regard for higher-order genomic structure (e.g., coding sequence). For each training set, samples from individual genomes were concatenated end-to-end to produce single DNA sequences to train a VLMC model.

Training a VLMC model was performed using the context algorithm [18,22], based on a previously developed data compression technique [23], which requires a single parameter, K. A larger value for K results in more pruning of a VLMC-derived tree, which leads to a less complex tree, and thus a model of smaller dimension. To determine an optimal value for K, we applied an approach similar to that of Mächler [18]. In brief, initial values of K (0, 0.5, 1.0, 2.0, 5.0, 10.0, and 15.0), termed K_0_, were used to create multiple VLMC models. For each K_0_, pruned VLMC models were used to emit *n*+1 base pairs. The first 10,000 base pairs were discarded to allow the simulation model to stabilize. Subsequent VLMC models were created for values of K from 1 to 20 in increments of 0.1 and used to predict the (*n*+1)^th ^base pair from the initial VLMC output. This process was iterated 1,000 times for each value of K_0_, and the number of correct predictions was recorded.

**Figure 1:**
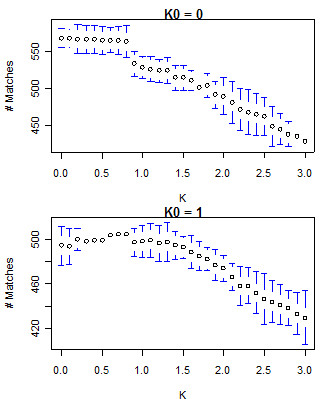
**VLMC next-base prediction accuracy for K_0 _= 0 and K_0 _= 1 using the 500 bp sampling strategy**. Error bars represent the 95% confidence interval around next-base prediction accuracy across 5 iterations.

Bootstrapping with multiple values of K_0 _revealed a plateau maximum accuracy of *n*+1 for values of K between 0 and 0.75, as shown in Figure 1. K = 0.75 was selected as the value for the pruning parameter to balance both overall accuracy as well as model parsimony. VLMC models, trained with K = 0.75, were generated using the sampling strategy described above. These VLMCs were used to generate an initial set of 100,000 25-mer probes. These probes were screened for a melting temperature, T_m_, between 58° and 68° C and a calculated free energy of self hybridization (ΔG, calculated using UNAFold [24]) greater than -1.1. Melting temperature calculations were carried out using the Primer3 software package [25]. In addition, probes with mono-runs of guanine bases longer than three were eliminated due to their propensity to form g-tetrads or pseudo-knots [26,27] which limit their availability for hybridization. The remaining probes were ranked by decreasing ΔG of self-hybridization, and the top 12,900 probes from each K set were selected. In addition to the three VLMC-derived sets of probes, a set of random probes was generated for comparison. 100,000 unique 25-mer DNA sequences were created from a uniform nucleotide distribution. This set of random sequences was then put through the same filtering and ranking process as the VLMC-derived probes, and the top 12,900 random probes were selected.

**Figure 2:**
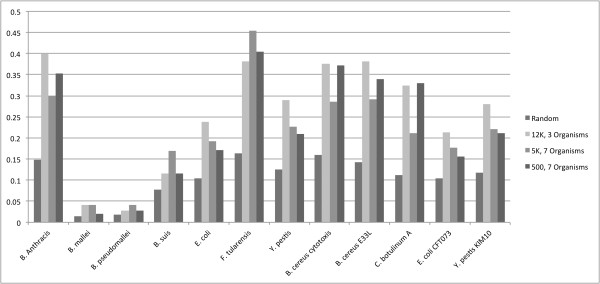
**Specificity in Hits/Kilobase of the VLMC trained vs. random probes against a panel of gram negative and positive prokaryotic organisms**. Specificity is defined as the number of times each probe is aligned to the target genome.

Finally, to evaluate the specificity of the random and VLMC-derived probes, we aligned each set of 12,900 25-mer probes against a panel of twelve Gram-positive and -negative prokaryotic organisms. This set consisted of the seven organisms used to train the VLMC, plus five additional genome sequences (*B. cereus *cytotoxis, NC_009674; *B. cereus *E33L, NC_006274; *C. botulinum *A, NC_009697; *E. coli *CFT0703, NC_004431 and *Y. pestis *KIM10, NC_004088). Alignment of probes to each genome was performed with *segemehl *[28], an algorithm designed for the alignment of short reads from next-generation sequencing experiments with support for insertions and deletions. For each organism, we calculated the specificity of each probe, defined as the number of times the probe aligned to the target genome per kilobase of genomic sequence ("Hits/KB'). As seen in Figure 2, the VLMC-derived probes have at least a 1.5, and an average of 2.1, fold increase in rate of alignment to each organism when compared to random probe sequences. The set of probes generated by sampling 500 base pairs, shown to perform slightly better in *n*+1 prediction by bootstrapping than that by 5000 base pairs, was selected to create a microarray for experimental testing. Of the top 12,900 probes, 18% were randomly duplicated for quality control purposes. The resulting 15,200 probes were sent to Agilent Technologies (Santa Clara, CA) for synthesis on their 8 × 15 K Custom Array format.

### Microarray Hybridization

To hybridize against the VLMC-derived probe set and generate data, the purified genomic DNA from 3 different simulant strains: *Bacillus cereus *(BC), *Bacillus subtilis *(BS) (as within-genera stand-ins for *B. anthracis*), and *Pantoea agglomerans *(PA) (as a gram-negative stand-in for *Yersinia pestis*), was fragmented and amplified using a Sigma GenomePlex^® ^Whole Genome Amplification (WGA) kit. 10 ng of purified genomic DNA was randomly fragmented using the WGA kit to yield fragment lengths of 75 - 1500 base pairs with an average fragment length of 400 base pairs. Fragmented DNA was then flanked by universal priming sites and amplified through 14 rounds of PCR. Amplified DNA was precipitated using 1/10 volume of 3 M sodium acetate (pH 5.2) and 2 volumes of 100% pure ethanol at -80°C for 2 hours. DNA was fluorescently labeled by reacting with the N7 of guanine using the with ULYSIS Alexa Fluor^® ^546 Nucleic Acid Labeling kit (Invitrogen). Excess dye was removed with an Agilent Genomic DNA Purification Module spin kit. Samples were then concentrated to 250 ng of DNA in 7μl. Labeled DNA was prepared for hybridization with 4.5μl Agilent 10 × GE Blocking Agent and 22.5μl Agilent 2 x CGH Hybridization buffer using an Agilent Oligo aCGH Hybridization kit. Samples were denatured at 95°C for 3 minutes followed by 30 minutes at 37°C. 11μl of KreaBlock was added to each sample to reduce background fluorescence. 40μl of prepared sample was then loaded onto Agilent 8 × 15 K Custom Arrays which were hybridized for 16 hours at 42°C. Arrays were washed (Agilent Oligo Wash Buffer Kit) for 5 minutes and then scanned on a Molecular Devices GenePix 4100 A scanner. Feature extraction was performed using Agilent's Feature Extraction software v9.5.3.1 and samples underwent quantile normalization via the Bioconductor *limma *package [29] in R.

Ten technical replicate arrays were generated for each of the three simulant species resulting in a total of 30 arrays for training and validation of the detection model (Table 2). Spike-in samples consisting of short oligos designed to bind to specific probes of the array were used as a positive control. Two spike-in arrays were run for each of two different concentrations to determine an optimum: 1% and 0.1% of total DNA concentration. Spike-in was then added at a 1% concentration to each single species array. Finally, 8 mixed samples were prepared based on 4 possible combinations of three single genomes (2 arrays per combination) in equal ratio for a total of 250 ng per array (Table 2). The mixed samples were labeled as: BC/BS/PA_1, BC/BS/PA_2, BC/BS_1, BC/BS_2, BS/PA_1, BS/PA_2, BC/PA_1, and BC/PA_2.

**Table 2:** Experimental Design

Genomic DNA	# Arrays	gDNA
B. subtilis	10	250 ng

B. cereus	10	250 ng

P. agglomerans	10	250 ng

B. subtilis/B. cereus	2	125 ng/species

B. cereus/P. agglomerans	2	125 ng/species

B. cereus/B. subtilis/P. agglomerans	2	125 ng/species

Oligo spike-ins	2	2.5 ng and 25 ng

Whole genome amplified DNA mixture concentrations were used to generate array data.

### Detection Model

A multivariate mathematical model based on partial least squares regression (PLSR) was developed to capture the signature of each simulant organism. Briefly, given a number of predictors, or independent variables, PLSR iteratively finds the best fit for one or more response, or dependent variables by maximizing the correlation between the two variables [30,31]. PLSR seeks to maximize correlation between the response and predictor variables while capturing and explaining most of the variation within the covariate space by constructing new predictor variables, or latent variables, as linear combinations of the original predictor variables.

In this study, the covariate matrix, X = (x_1_,...,x_*m*_), is a (*n *× *m*) matrix of *n *= 12,900 observations and, *m *= 4 predictor variables. Each variable, x_*j*_, for *j *∈ {1,2,3}, represents the vector of hybridization values, *x_ij_, i *= 1,...,*n*, averaged over 10 replicate arrays for the *j*^th ^simulant species (see Table 2), and x_4 _represents that of the oligos averaged over two arrays (see Table 2). The response matrix, Y = (y_1_,...,y_*s*_), is a (*n *× *s*) matrix of *s *= 8 dependent variables representing 4 possible combinations of the three simulant organisms, with two replicate arrays for each combination, hybridized against the probe set. Both the predictor and response matrices were then standardized (mean-centered and scaled) before analysis was performed.

The predictor and response matrices are decomposed into the following forms:(1)

where T and U are the respective (*n *× *h*) score matrices of *h *latent variables, *n *≤ *s*; P^*T *^and Q^*T *^are the respective (*h *× *m*) and (*h *× *s*) transpose matrices of loadings, and E and F are (*n *× *m*) and (*n *× *s*) matrices of residuals. We used a variation of PLSR called SIMPLS algorithm [30] to iteratively find the latent vectors that best explain the relationship between X and Y matrices, by simultaneous decomposition of the two matrices. A diagonal matrix of regression coefficients, B, is estimated as the normalized inner product of the two score matrices, which describes the inner relationship between the predictor and response variables:(2)

To determine whether a simulant organism is present in a mixed sample, and the amount of its contribution to the sample, a (*m *× *s*) matrix of weights was estimated based on the diagonal matrix B (see equation (2)) and the loading matrices of the predictor and response variables:(3)

The goodness of fit of the model for each test sample was determined using the *R*^2 ^statistic, which is the normalized value of the total squared error explained by the model. Finally, to determine which probes are critical in differentiating between patterns of hybridization of the simulant species, the contributing value of each probe to the goodness of fit was assessed using the Hotelling's *T*^2 ^statistic [31], a statistical measure of the multivariate distance of each observation score from the center of the observations per probe:(4)

where *k *is the number of sample observations per probe, T_*i *_is the vector of *k *sample observation scores in row *i*, for *i *= 1,...,*k, μ_i _*is the mean value of *k *observation scores in row *i*, and S^-1 ^is the inverse of the sample covariance matrix. All scripts were written in Matlab 7.6.0 (R2008a).

## Results

### Signal Detection

The first three latent variables from the PLSR model, *h *= 3, achieved maximum correlation with the response variables while together they captured most of the variation in the predictor matrix (>86%) and response matrix (>74%). Thus, the PLSR model was calibrated using the first three components to build a predictive model of the response matrix.

**Figure 3:**
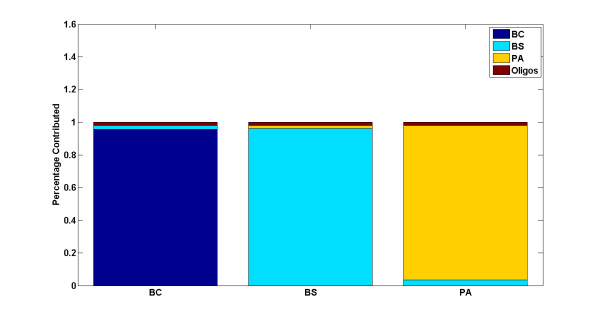
**Validation of the PLSR model using single species arrays by iterative leave-one-out**. All three organisms are classified with high *R^2 ^*values.

**Figure 4:**
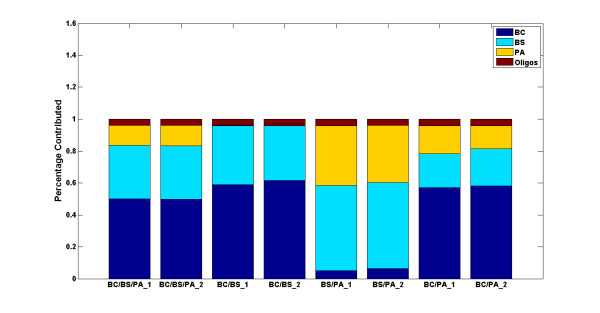
**Capturing the DNA signature of single species in mixed samples**. The PLSR model was tested on mixed samples and the presence of all contributing organisms was correctly detected.

The PLSR model was first validated using the training data on single species arrays by iterative leave-one-out cross validation. In each round of iteration, one array, from the set of 30 single species arrays, was randomly selected as a test sample and excluded from the training phase. The model was then trained on the remaining 29 arrays and the two oligo spike-in arrays, and tested on the array that was left out. Equation (3) was used to predict the outcome of each round of experiment, namely the amount of contribution of each simulant organism to the test array. These experiments were repeated 200 times and the average value was reported as the final predictive value. As illustrated in Figure 3, all three simulant organisms were classified correctly with high specificity (mean(*R^2^*)) = 0.97, CI = 0.95). The percentage of contribution as depicted on the y-axis represents the specificity or amount of contribution of each organism to the test sample as explained by the model. To test its predictive power, the model was trained on 4 predictor variables consisting of the three simulant species and oligo spike-ins, representing the X matrix (see equation (1)), and tested on 8 mixed samples, representing the Y matrix. As depicted in Figure 4, the signature of all contributing individual organisms in each mixture was captured correctly in all 8 samples, leading to a 100% true positive rate, or sensitivity, of the model (mean(*R*^2^) = 0.76, CI = 0.95). In two BCPA samples (the last two stacked bars in Figure 4), however, the signature of the third organism, BS, was incorrectly detected, leading to a 6.25% false positive rate, or 93.75% specificity. This is because 2 out of 8 samples report the presence of one additional organism out of four possible contributing organisms: (2/8)(1/4) = 1/16 = 0.0625.

**Figure 5:**
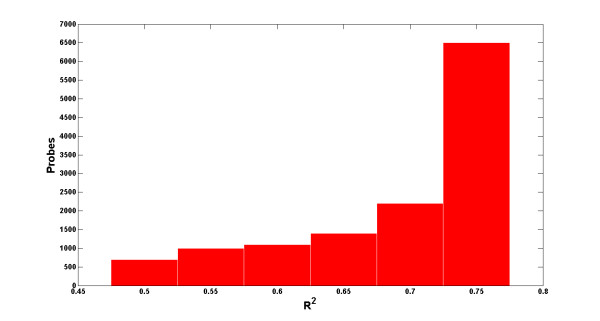
**Distribution of average goodness of fit**. For each mixed sample, probes are sorted in decreasing order of their *T^2 ^*statistic and iteratively added to the model; the *R*^2 ^statistic is computed until all probes are included. The average *R*^2 ^statistic across all mixed samples is recorded at the end of each iteration.

To determine the contribution of each probe to the goodness of fit of the model, probe values were assessed using the Hotelling's *T^2 ^*statistic (see equation (4)). For each mixed sample, probes were sorted in descending order of their *T^2 ^*statistic. The PLSR model was then run iteratively, each time and for each mixed sample, adding the next top 100 probes and computing the *R^2 ^*value up to that point until all 12,900 probes were included in the model. At the end of each iteration, the average value of the *R^2 ^*statistic of all samples was recorded. Figure 5 illustrates the distribution of *R^2 ^*statistic as a function of number of sorted probes included in the model.

### Sparse Sampling and Signal Detection

The distribution of probes in Figure 5 suggests that a relatively small subset of probes may be sufficient to generate, and differentiate between, the hybridization patterns that signify the genomic imprints of the three single species. In Figure 5,  of the overall average *R^2 ^*statistic is achieved using only about 700 probes, while using an additional 6,500 probes contributes only about 5% to the average *R^2 ^*value (the rightmost bin in Figure 5). To test the hypothesis that a smaller set of probes is capable of accurately capturing the signature of each organism, increase the detection specificity, and thus reduce the false positive rate observed in the previous section (Figure 4), the following sparse sampling algorithm was designed:

1. For each mixed sample

a. Probes were sorted in decreasing order of their *T^2 ^*values.

b. Probes with high *T^2 ^*values were selected for further investigation, if their value was greater than *μ*_*T*^2 ^_+ *cσ*_*T*^2 ^_, where *μ*_*T*^2 ^_and *σ*_*T*^2 ^_are the respective mean and standard deviation of the sample *T*^2 ^values, and *c *is a scalar.

2. Probes with high *T*^2 ^values shared by four out of eight samples (or two out of four combination groups) from step 1.2 were selected as the final set for PLSR analysis.

**Figure 6:**
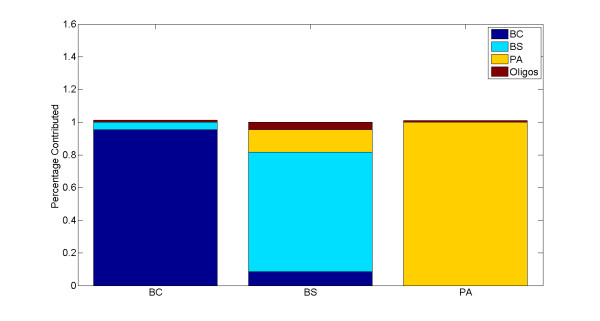
**Validation of PLSR**. Validation performed using 47 probes with iterative leave-one-out. All three stimulant organisms were classified correctly with high *R^2 ^*values.

The PLSR model was then run on data collected on the final probe set. For the scalar values 2 ≤ *c *≤ 4.35, the size of the probe set varied from 47 to 185. In all cases, the model was capable of accurately capturing the signature of single organisms in the mixed samples while the false positive rate was significantly reduced. Here, we demonstrate the results for *c *= 4.35, which generates the smallest probe set consisting of 47 probes capable of capturing the DNA signature of the simulant organisms while achieving a significantly diminished false positive rate. Figure 6 illustrates the result of the validation phase, where all three simulant organisms are classified correctly with high specificity (mean(*R^2^*) = 0.97, CI = 0.95).

**Figure 7:**
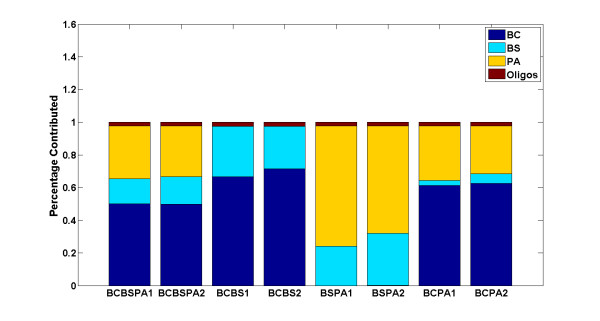
**Capturing the hybridization signature of single species in mixed samples**. The PLSR model was tested on mixed samples and the presence of all contributing organisms was correctly detected.

To test the predictive power of the model using the final set of 47 probes, the PLSR was then tested on the eight mixed samples. As depicted in Figure 7, the signature of the single species was accurately captured in each mixture leading to a 100% true positive rate, or sensitivity, of the model (mean(*R*^2^) = 0.77, CI = 0.95). Note that the observed false positive in the two BCPA samples of Figure 4 when all 12,900 probes were used, is greatly diminished when the model is run using 47 probes.

### Sparse Sensing

In this section we demonstrate that, in retrospect, the sparse sampling algorithm, developed in the previous section, closely follows the principles of compressive sensing when the matrix of intensity values is properly mapped to generate a sensing matrix. Recall the main condition of CS--that for a signal to be compressively sensed, it must be sufficiently sparse (*K*-sparse). Here, the target vector, **x**, has only three non-zero elements, namely the concentrations of the three simulant organisms in captured samples and the remaining *N*-3 entries are zero. Because in principle, the number of potential organisms in a location at a point in time, *N*, is very large, **x **is considerably sparse (*K *= 3). The vector of *M *measurements, **y**, consists of 12,900 intensity values for each mixed sample. The key challenge in the application of sparse sensing is in the design of the sensing matrix that satisfies the RIP and results in accurate recovery of **x **using the matrix notation y = Φx. It has been shown that sparse binary random matrices satisfy the RIP [17]. Here, we show how the results of our sparse sampling algorithm can be mapped to a sparse binary random sensing matrix that together with the hybridization measurements uniquely identifies the presence of each simulant organism in the mixed samples.

**Figure 8:**
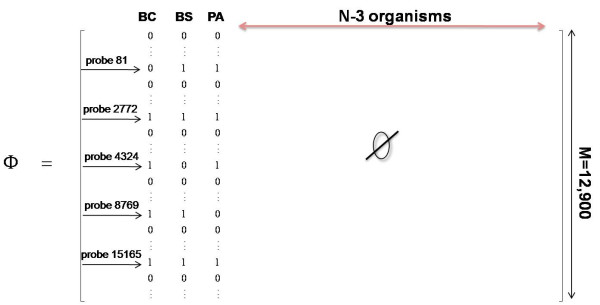
**Schema of the sensing matrix**. The probes as indicated by their row numbers on the left of the matrix belong to the set of 47 selected probes.

Let *S *denote the set of 47 selected probes generated by the sparse sampling algorithm. Define *I*(*i, j*) as the intensity (hybridization) value of the *j*^th ^organism (column *j*), against the *i*^th ^probe, *i *= 1...*M *and *j *= 1...*N*. Let *μ_i _*denote the mean of the intensity values in row *i*, and *φ_ij _*be the (*i, j*)^th ^element of the sensing matrix. Then we have:

The above mapping results in a very sparse, random binary matrix. The structure of the sensing matrix is presented in Figure 8, where the positions and binary patterns of five out of 47 probes, covering seven possible binary combinations, are shown as examples. A "1" entry in the (*i, j*) position of the matrix indicates that the organism in column *j *has a relatively large intensity value when hybridized against the *i*^th ^probe, and thus is present in the mixed sample in question. Similarly, a "0" entry indicates that the organism is not present in the mixed sample. Specific binary patterns uniquely correspond to a group of mixed samples. For instance, all rows with the binary pattern "101" map to a set of unique probes, not shared by other binary patterns, against which BC and PA are hybridized at a relatively high value but not BS. This pattern corresponds to the last two mixed samples in Figure 7. The vector of hybridization measurements, **y**, then consists of non-zero intensity values that correspond to each binary combination in rows pertaining to the final 47 probes.

**Figure 9:**
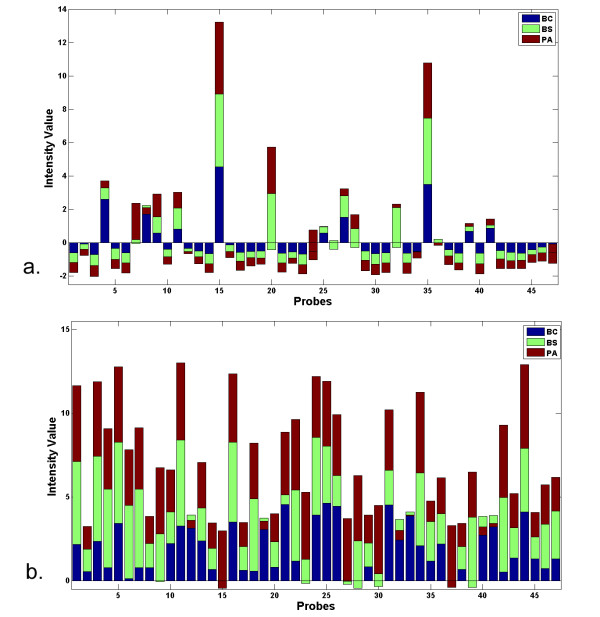
**Bar plot of intensity values**. (a) A set of 47 randomly selected probes; (b) The final set of 47 probes selected by the sparse sampling algorithm.

Finally, the distribution of the intensity values derived from hybridization against the 47 selected probes was compared to the average distribution of 500 runs of 47 randomly selected probes. The respective mean and standard deviation of the intensity values for the set of 47 selected probes were 2.02 and 0.82, while the average of those for 500 randomly selected sets of 47 probes were 0.003 and 0.72. The difference in the respective standard deviation values is not large, yet, the dichotomy is most apparent when the mean values are compared. As a result, the coefficient of variation (standard deviation divided by mean) for the 47 selected probes is 0.41, indicating close concentration of intensity values around the mean, and that of the randomly selected probes is 271.6, indicating large dispersion of intensity values with respect to the mean. Figure 9 (a, b) are the respective bar plots of the intensity values of the three single species hybridized against an instance of a set of 47 randomly selected set of probes and those of the 47 selected probes.

## Discussion

It is well understood that in spite of vast amount of shared sequences among biological organisms, most comprise unique sets of oligomers based on which they can be differentiated at various biological scales. This critical finding has enhanced the ability to design microarray-based biosensors capable of detecting multiple biological agents whose signatures are included in the array. As more viral and bacterial species are sequenced and their DNA signatures are retrieved, microarray scalability presents a challenge to the design of target-specific biosensors. At the same time, such a targeted approach to biosensing is ill-equipped when a biological threat is due to the presence of an agent whose signature is not considered in the microarray design either because it was outside the realm of expectation (e.g., previously eradicated but re-emerging pathogens) or is unknown (e.g., newly emerging strains or an engineered pathogenic sequences). An open system approach to biosensing is a new concept. If properly designed, an open system biosensor can address the aforementioned challenges from which conventional biosensors suffer.

The equivalence of our sparse sampling algorithm and compressive sensing in the context of open-target sensing has important implications for biosensing. First, that the genomic imprints of biological organisms can be represented in a compressed format, and thus a relatively small DNA microarray can be used to decode the signature of multiple organisms in mixed, and potentially complex environmental samples. Second, that the sparsity condition likely applies to environmental sampling and detection of biological events, and thus the cost and size of the array can be kept in check. And third, that the previously un-encountered microorganisms can be detected if they are present in the environment at sufficient concentrations, even though their unique DNA sequences are not explicitly accounted for in the array design.

Two potential limitations of this study must be addressed for future consideration. First, despite relatively extensive laboratory experimentations performed for this study, the number of biological organisms tested and selected to generate mixed samples is small. To demonstrate the utility, efficiency, and robustness of an open system approach to biosensing, a greater spectrum of biological agents must be tested and their hybridization patterns evaluated against the microarray probes.

Second, with respect to the probe design a set of evaluations were performed to select the final design of the probe set, where the specificity of the randomly generated and VLMC-derived probes were compared by aligning each set of 12,900 25-mer probes against a panel of twelve Gram-positive and -negative prokaryotic organisms (Figure 2). While the specificity of all three VLMC-derived probe sets was substantially higher than that of random probe sequences, the average performance of the three VLMC-derived sets of probes is relatively the same across all organisms. It is important to note, however, that we only generated one set of probes for each sampling strategy. In principle, the average outcome of multiple runs of simulations is required to arrive at statistically significant results. We selected the first sampling strategy, a random sampling of 500 bp from each of the seven pathogenic sequences, for designing the final probe set based on its slightly higher prediction accuracy than those of the two probe sets generated using the competing sampling strategies. A more comprehensive examination of these and other sampling strategies are needed to determine which strategy, or set of strategies, leads to the best probe sequences design for differentiating between the DNA signatures of multiple organisms.

## Conclusions

In this paper, we hypothesized and demonstrated that a relatively small non-specifically designed DNA microarray was capable of identifying the presence of three test organisms in mixed DNA samples with high sensitivity and specificity without specifically targeting these organisms. Coupled with a multivariate detection model and sparse sampling of the microarray probes our prototype open-target biosensor was demonstrated to follow the design principles of CS.

Three observations are worthy of note here, and should also be considered in future work. First, sparse sampling of 12,900 probes, based on a two-layer filtering, led to the selection of the smallest set consisting of 47 probes capable of accurate identification of three simulant organisms in the mixed samples. This resulted in a considerable reduction in the array size, based on which a sparse, binary, random sensing matrix was designed. However, our goal was not to derive the minimum number of probes required to differentiate across three test organisms in mixed DNA samples, but to demonstrate the feasibility of designing a small DNA microarray for 'open-target' sensing of multiple organisms and applicability of sparse sampling to biosensing. It remains uncertain whether a mathematical function can be formulated that describes the relationship between the number of organisms to be sensed and the size of an 'open-target' microarray.

Second, qualitative examination of the relationship between the size of the array and its detection specificity uncovers an important difference between 'open-target' and 'target-specific' microarray-based sensing platforms. In 'target-specific' sensing, as the size of the microarray is increased to include molecular signatures of newly sequenced organisms, the false-positive rate is expected to decrease, or equivalently the specificity is expected to increase. In 'open-target sparse sensing', the false-positive rate approached zero, or equivalently the specificity reached 100%, as the size of the array was substantially reduced by pruning the less informative probes. This observed dichotomy between 'open-target' and 'target-specific' sensing with respect to the relationship between the array size and detection specificity, while promising, will have to be further validated in future studies.

Third, the distribution of the intensity values of the final set of 47 selected probes is qualitatively different than that of the average of 500 runs of 47 randomly selected probes (see Figure 9). The sparse sampling algorithm was applied to 12,900 probes without any constraint imposed on probe selection except that a selected probe would have a high *T*^2 ^value. Indeed, the application of sparse sampling algorithm resulted in the selection of high *T*^2 ^probes which captured the difference in the hybridization patterns of BC and BS, and greatly reduced the false positive rate previously observed (compare Figures 4 and 7). This finding should be more closely examined by testing more organisms and by the sequence alignment of each selected probe against the genomic sequence of each organism.

To our knowledge, this is the first study to introduce an 'open-target' approach to DNA microarray based biosensing, and demonstrate a proof of concept through three elements of probe design, laboratory data generation, and mathematical modelling. Future directions of this work include improvement to the probe design as guided by the analysis and experiments, expansion of the reference library to encompass additional test organisms, and environmental testing by external air sampling to provide a more realistic and complex environmental background.

## Competing interests

The authors declare that they have no competing interests.

## Authors' contributions

MM performed the mathematical analysis and drafted the manuscript. DKW performed the array design, initial bioinformatic analysis, and assisted with drafting the manuscript. MWP contributed to the bioinformatic analysis and assisted with drafting the manuscript. HBS led laboratory method development and execution.

FNS contributed to the laboratory experiment execution. JCD directed the study and assisted with drafting the manuscript. All authors read and approved the final manuscript.
